# Surface treatments for controlling corrosion rate of biodegradable Mg and Mg-based alloy implants

**DOI:** 10.1088/1468-6996/16/5/053501

**Published:** 2015-09-08

**Authors:** M S Uddin, Colin Hall, Peter Murphy

**Affiliations:** 1 School of Engineering, University of South Australia, Mawson Lakes, SA 5095, Australia; 2 Mawson Institute, University of South Australia, Mawson Lakes, SA 5095, Australia

**Keywords:** Mg/Mg-based alloys, biomedical implants, corrosion resistance, biodegradability, surface treatment, coating, mechanical processing

## Abstract

Due to their excellent biodegradability characteristics, Mg and Mg-based alloys have become an emerging material in biomedical implants, notably for repair of bone as well as coronary arterial stents. However, the main problem with Mg-based alloys is their rapid corrosion in aggressive environments such as human bodily fluids. Previously, many approaches such as control of alloying materials, composition and surface treatments, have been attempted to regulate the corrosion rate. This article presents a comprehensive review of recent research focusing on surface treatment techniques utilised to control the corrosion rate and surface integrity of Mg-based alloys in both *in vitro* and *in vivo* environments. Surface treatments generally involve the controlled deposition of thin film coatings using various coating processes, and mechanical surfacing such as machining, deep rolling or low plasticity burnishing. The aim is to either make a protective thin layer of a material or to change the micro-structure and mechanical properties at the surface and sub-surface levels, which will prevent rapid corrosion and thus delay the degradation of the alloys. We have organised the review of past works on coatings by categorising the coatings into two classes—conversion and deposition coatings—while works on mechanical treatments are reviewed based on the tool-based processes which affect the sub-surface microstructure and mechanical properties of the material. Various types of coatings and their processing techniques under two classes of coating and mechanical treatment approaches have been analysed and discussed to investigate their impact on the corrosion performance, biomechanical integrity, biocompatibility and cell viability. Potential challenges and future directions in designing and developing the improved biodegradable Mg/Mg-based alloy implants were addressed and discussed. The literature reveals that no solutions are yet complete and hence new and innovative approaches are required to leverage the benefit of Mg-based alloys. Hybrid treatments combining innovative biomimetic coating and mechanical processing would be regarded as a potentially promising way to tackle the corrosion problem. Synergetic cutting-burnishing integrated with cryogenic cooling may be another encouraging approach in this regard. More studies focusing on rigorous testing, evaluation and characterisation are needed to assess the efficacy of the methods.

## 1. Introduction

With the tremendous development of biomaterials over the decades, medical implantation in orthopaedic, cardiovascular and paediatric applications have become one of the most successful surgical procedures, and offer solutions to diseased or damaged bone and narrowed coronary arteries. For instance, bone fractures account for about 54% of injury hospitalisations in Australia [1] and the number is increasing across other parts of the world due to an aging population [2, 3]. In order to fix bone fractures, currently, permanent metallic implants made of stainless steel, titanium, and cobalt-chromium alloys are used. Because of their high strength and good corrosion resistance, they have been widely used as the load-bearing implants for bone healing and repair of damaged tissues [4–6]. The key problems with these permanent implants are two-fold. Firstly, they cause stress shielding [7], which is the reduction of load borne by the bone, by the implant which results in osteopenia [8]. This effect is caused by a distinct mechanical mismatch in elastic modulus, between the implant (100–200 GPa) and the surrounding bone (10–45 GPa) [9]. This further weakens the bone–implant interface, implant loosening and impairment of regular bone healing and nearby anatomical structures. Secondly, after the tissues heal sufficiently for a certain period of time, permanent metals must be removed from the body by using a secondary surgical intervention. Additional surgery causes an increase in costs to the health care system, as well as emotional stress to the patient.

In order to solve the problems with permanent metallic implants mentioned above, over the years, new Mg/Mg-based alloy implants have been studied [9–11]. Such biocompatible implants can dissolve in biological environments, while providing sufficient mechanical integrity and support for a certain period of functional use. In addition to their biodegradability, the alloys exhibit a higher yield strength and a lower Young’s modulus of elasticity (40 GPa), which approaches that of natural cancellous bones (10–40 GPa). Such a good match in mechanical stiffness enables Mg/Mg-based alloys to be adopted to minimise stress shielding when applied in orthopaedic implants. Further, being an essential element and present in ample quantities in the human body, Mg ions facilitate metabolic reactions and various biological mechanisms [12]. The aforementioned factors all help bone tissue to grow, and improve the bone healing process while maintaining a suitable mechanical integrity.

However, the key challenge faced by Mg/Mg-based alloys, which prevents their successful clinical use, is their rapid corrosion in biological environments (i.e. saline media). Such rapid corrosion causes hydrogen gas generation at a rate which is too fast for the bone tissue to accommodate, hence degrading its mechanical integrity before the bone heals completely. Therefore, it is crucial to address the above issues to develop Mg/Mg-based alloys with improved corrosion performance before their implantation in the underlying physiological environments.

Over the years, researchers have studied different methodologies and approaches to control the corrosion rate, most importantly, to delay the degradation of the alloy at a rate that matches bone healing [9, 13–16]. Notable approaches are the addition of other alloying elements, the application of protective thin films, and mechanically induced stress. By conducting laboratory, *in vitro* and *in vivo* tests, the corrosion performance, along with biocompatibility and biodegradability of the processed alloys, has been assessed. All strategies reveal improvements in controlling the *in vitro* the corrosion rate, to some extent. However, the improvement is not adequate to heal the bone sufficiently and hence still prevents the successful use of the alloys in orthopaedic applications, except for a few preliminary trials, as outlined in [17]. It is reported that among all approaches, surface treatments exhibit the most promising performance improvements, and are thus potentially becoming a growing subject of research in Mg-based orthopaedic implants.

This paper presents a comprehensive review of surface modification techniques adopted by researchers over the last decade to improve the corrosion resistance of biodegradable Mg/Mg-based alloys. The main aim is to perform a critical review and analysis of the approaches including their advantages and disadvantages, effects of key process parameters on the corrosion rate, including microstructure, mechanical integrity, and biocompatibility. The outcome of this study will identify future challenges in the area, and urge us to develop potentially enhanced biodegradable Mg-based alloys which can be successfully applied as orthopaedic and cardiovascular implants.

## 2. Biodegradable Mg/Mg-based alloys as implant materials

### 2.1. Application in orthopaedics

Bone fractures are found to be the primary cause of injury hospitalisations across the world. They cause misalignment of normal bone orientation, resulting in dysfunction. Medical surgeons then attempt to reproduce the normal anatomy of fractured bone using implants. Conventionally, for bone fracture repair, metallic implants, e.g. bone screws, nails, and plates, are used. While they have good biocompatibility and mechanical strength, their elastic modulus is significantly larger than the surrounding bone, causing stress shielding. Once the bone heals sufficiently through osteointegration, implants need to be taken out of the body through a secondary surgery after a certain period of time (1–2 years).

Magnesium (Mg) is essential to human metabolism and is the fourth most abundant cation in the human body, with an estimated 25 g of Mg stored in the human body and approximately half of it contained in the bone tissue. In addition, Mg is a cofactor for many enzymes, which stabilises the structure of DNA and RNA. Mg exhibits fast corrosion in the chloride-containing physiological environment. The above made Mg a biodegradable and biocompatible material for potential use in orthopaedic and trauma surgeries. Initially introduced by the Australian-German physician Erwin Payr, Mg has been used for recovering joint motion for bone fracture fixation with wires and pegs as intramedullary rods [10]. Later on, the use of Mg sheets was studied for restoring joint motion in animals and humans.

It is reported that if the surface area of a Mg implant is less than 9 cm^2^, the dissolved Mg^2+^ would be easily consumed by the human body. However, the rapid generation of hydrogen/hydroxide anions due to the corrosion process could pose serious problems for patients. For instance, such severe side effects of rapid generation of hydrogen may prevent the possible application of a Mg stent in a blood circulating vascular system [18]. Therefore, the rapid degradation of pure Mg due to accelerated corrosion in a physiological environment is found to be the main concern and prevents the use of Mg as an implant in bone or joint repair procedures. This forced medical surgeons to move forward with highly corrosion-resistant hard metallic devices [19–21].

**Figure 1.**
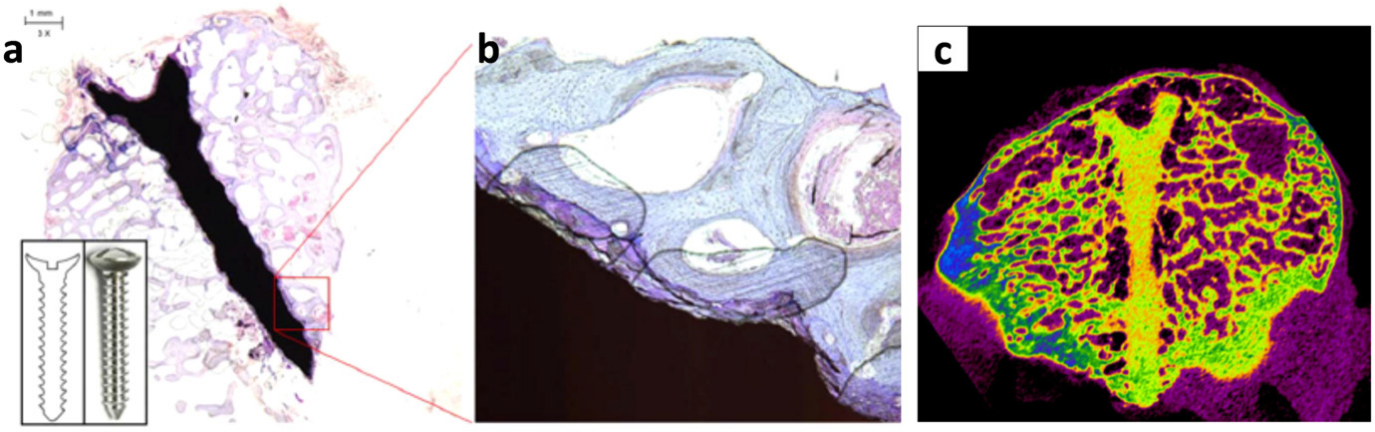
(a) Histology of extruded Mg5-wt%Ca-1wt%Zn alloy bone screw in femoral condyle (b) magnified view of cross section of the bone screw, (c) micro-computed tomography of the bone screw at 24 weeks after operation. Reprinted by permission from Macmillan Publishers Ltd: [22], copyright 2013.

In order to overcome the challenge faced by pure Mg, researchers have explored the potential opportunity of improving corrosion performance by alloying various appropriately selected elements into Mg. For instance, metals such as zinc (Zn), aluminium (Al), silver (Ag), yttrium (Y), zirconium (Zr), neodymium (Nd) and manganese (Mn) have been used as alloying elements to enhance mechanical properties and corrosion behaviour. Mg–Ca, Mg–Ca–Zn and Mg–Zn are a few examples of Mg-based alloys. Cha *et al* implanted and observed a bone screw made of as-extruded biodegradable Mg-5.0Ca-1.0Zn alloy on the femoral condyle of a New Zealand white rabbit for 24 weeks [22]. Histological and micro-computed tomographic analysis shows new bone formation and bone modelling with reduced bubble formation and foreign body response around the progressively degraded implant sample, as can be seen in figure 1. With the proper selection of alloying elements and their compositions, the microstructure of Mg material can be designed in such a way that the mechanical properties become similar to those of cancellous bone, hence making it ideal for use as a bone substitute [23]. Very recently, Farraro *et al* [24] conducted two case studies exploring the efficacy of Mg-based alloys in tissue engineering. The first case study used an interference screw made of Mg alloys (AZ31) for fixation of tissue auto-grafts during anterior cruciate ligament (ACL) reconstruction. First, a screw design was optimized using the finite element method, and ACL reconstruction on the goat stifle joint was performed to fix the autograft with the screw. Both *in vitro* and *in vivo* studies reveal an improved performance of the interference screw in terms of joint stability and graft function. The second study by the same authors [24] was focused on a novel Mg-based alloy (AZ31) ring to reduce the gap between the two torn ends of the ACL in order to serve as an internal splint as an alternative for mechanical augmentation. The function of the ring was evaluated *in vitro* using cadaveric goat stifle joints. Experimental results show that joint function is restored providing the required mechanical integrity in the early stages of ACL healing and hence minimizing the disuse atrophy of implanted sites. This allows the Mg-based ring to be resorbed with time, enabling the healing of ACL to remodel and strengthen.

By varying the Ca concentration and using different fabrication techniques, Li *et al* [25] developed pins made of Mg–Ca alloys, implanted them into left and right rabbit femoral shafts and monitored the degradation process for 1, 2 and 3 months. They compared the performance of this pin with respect to c.p. Ti pins (the control pin made of Ti supplied by AO Company; ‘c.p.’ is the model name of the pin). Initial cytotoxicity tests showed that the Mg-1.0Ca alloy did not induce any toxicity in the cells. Periodic observation of implanted pins revealed that Mg–Ca alloy pins degraded gradually during the test period as was observed by the reduction of pin diameter. Further, new bone was found to be formed around the Mg–Ca alloy pins, whereas no noticeable bone growth was observed around c.p. Ti pins, indicating superior mechanical integrity and osteogenesis of Mg–Ca alloy pins around the bones.

In a similar study, Thomann *et al* [26] investigated the effect of alloying of Mg on the corrosion process, in which Mg was alloyed with Ca (Mg-0.8Ca) and with aluminium and rare earth elements. After implanting the alloy into the marrow cavity of the tibiae of a white rabbit for 12 months, the Mg-0.8Ca alloy was found to show a strong bone–implant interface while revealing an average loss in the cross section of the implant. The progressive degradation of the implant (in volume) was 11%, 31%, and 51% after 3, 6, and 12 months, respectively.

Mg alloys containing Zn and Mn elements (e.g. Mg–2Zn–0.2Mn and Mg–1.2Mn–1.0Zn) were studied by Zhang *et al*, e.g. [23]. The alloys showed satisfactory mechanical properties and biodegradation performance. However, it was shown that Mn and Zn based alloys degraded relatively fast. For instance, a 9 week implantation of Mg–Zn–Mn based alloy revealed 10–17% degradation, while after 18 weeks the degradation of the same implant increased up to 54%.

### 2.2. Application in cardiovascular stenting

In addition to their potential application in bone fixation, Mg-based alloys have been considered as potential biodegradable coronary arterial stents. Following an angioplasty, coronary stenting is performed in order to open up and hold the narrowed artery to allow the normal blood circulation. The implantation has been considered a successful cardiovascular procedure in medical industries and is able to reduce restenosis, the phenomenon of re-narrowing of the artery after stenting.

While being successful in mediating coronary blood clots, currently available permanent metallic and bioabsorbable polymeric stents still face problems such as inflammation and the necessity of secondary surgical intervention. Polymeric stents are not able to provide sufficient mechanical strength to hold the stent open for a certain time as the blood vessel regains its original capability [27]. Clinical studies indicated that polymer stents also cause inflammatory reactions with the surrounding tissues of the artery. On the other hand, after implantation, metallic stents remain in the body and need further surgical intervention in case of potential complications such as damage to the blood vessel [28]. It is shown that, in response to the injury of the artery wall after implantation, metallic stents cause thrombosis and further thickening of inner lining of the artery [29]. Permanent metallic implants prevent the normal growth of the tissue of the artery, which is critical for children with implants in their bodies requiring stents to last for a lifetime [30]. Due to their presence in the body, permanent metallic stents often pose difficulties for further assessment of arteries by available medical technologies such as computer tomography (CT) and magnetic resonance imaging (MRI) [31]. The problems with metallic and polymeric stents thus pushed researchers to improve the treatment of narrowed or damaged coronary arteries. As an ideal solution to the above problem, it is expected that a stent will perform its function and provide the required support to restore the diseased artery, and then become absorbed by the body through a natural biological process.

In this regard, biodegradable metallic stents have received significant attention in the medical industry and are considered a potential alternative. Once implanted, the implant will degrade within the body while providing the required mechanical integrity, thus holding the blood vessel open until the vessel heals and redevelops completely. Over the years, researchers have studied the efficacy of biodegradable metallic stents, as outlined in a review article by Purnama *et al* [32]. There are two types of biodegradable metallic stents: iron-based and Mg-based alloys. The first iron-based biodegradable stent was fabricated from Armco^®^ iron (Fe > 99.8%) and implanted in the descending aorta of New Zealand white rabbits in 2001. The results showed no noticeable evidence of an inflammatory response, neointimal proliferation or toxicity in any organs [30]. However, a recent study by the same authors indicates that iron-based stents do not corrode completely during the long-term follow up period [33]. A faster degradation rate is thus essential for iron, which requires further studies focusing on the adjustment of the composition and the geometric design of the stent.

**Figure 2.**
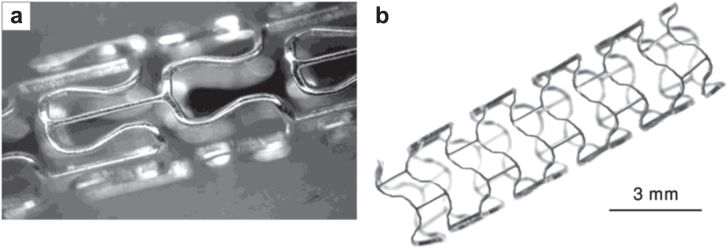
Lekton Magic coronary stent (a) non-expanded (reproduced from [37], copyright 2004 John Wiley and Sons), (b) expanded (reproduced from [33], copyright 2006 Cambridge University Press).

As an alternative to iron based stents, biodegradable Mg-based alloys have been used as superior candidates for cardiovascular stenting. For instance, the first human trial of a Mg-based metallic stent was conducted in PROGRESS-AMS (Clinical Performance and angiographic Results of Coronary Stenting with Absorbable Meta Stents) and the study showed a rapid degradation of the stent causing restenosis and loss of mechanical integrity [34]. In a similar study [35], the authors reiterated the fast degradation of Mg-based stents (i.e. in less than a month after implantation), while another study reported that Mg-based stents in a human body can be safely degraded after 4 months [36]. Di Mario *et al* [37] studied the Lekton Magic coronary stent (Biotronik, Bulach, Switzerland) made of WE43 magnesium alloy in the coronary artery of 33 mini-pigs. Figure 2 shows Lekton Magic stents in non-expanded and expanded states. Peeters *et al* [38] further investigated Lekton Magic coronary stents implanted in humans for treatment of lower limb ischemia of 20 patients, and reported that no symptoms of allergic or toxic reactions to the Mg-based stents were found. Given the safe application of the stent, a contradictory result in terms of reduced longer-term (6 months) performance of Mg-based stents was reported elsewhere [39]. This shows variability in the clinical data in terms the performance of Mg-based implants and thus warrants further research in this area.

Therefore, it is clear that fast degradation in the cardiovascular environment limits the performance of Mg-based alloy stents, and the challenge is how to regulate the degradation process. Research effort continues to further develop improved Mg-based stents in terms of design, material, and fabrication [40, 41].

## 3. Properties of Mg/Mg-based alloys

### 3.1. Microstructure

**Figure 3.**
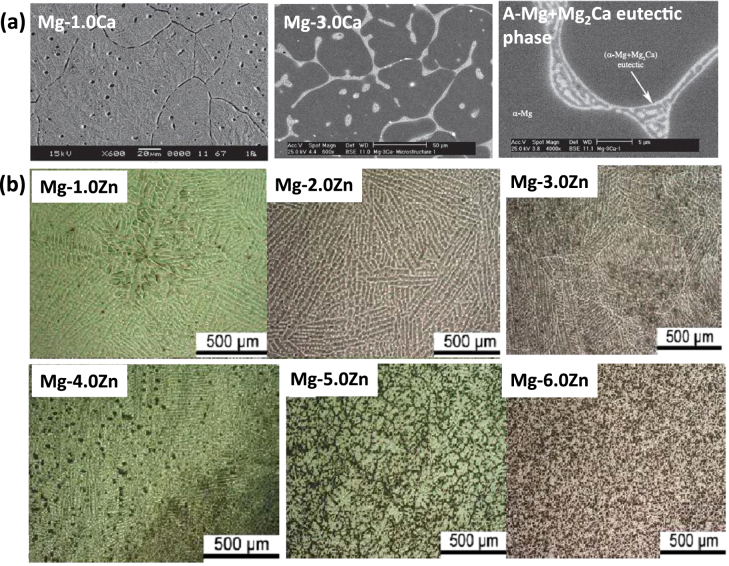
Microstructure of (a) Mg–Ca alloying (reproduced from [43], under CC BY-NC 4.0 license) and (b) Mg–Zn alloying (reproduced from [23], copyright 2011 Zhang B P, Wang Y, Geng L. Published in [23] under CC BY 3.0 license. Available from: http://dx.doi.org/10.5772/23929).

When pure Mg is alloyed with other elements, e.g. Ca, the underlying microstructures and phases formed define the mechanical properties of the alloy. Previous studies on a binary Mg–Ca alloy system revealed that, with a low concentration of Ca (less than 16.2%), the Mg–Ca alloy developed an alpha-phase solid solution and a eutectic structure (alpha-phase + Mg_2_Ca). Interestingly, Mg_2_Ca, as an intermetallic phase, was found to be electrochemically more active (representing the anodic role) than the alpha-phase and possess the same crystal structure as pure Mg [42], which enabled Ca to be considered a biodegradable alloying candidate. The addition of Ca thus enhances the corrosion resistance and keeps the grain size smaller. Further study showed that, with the increase of Ca content, the grain size and the dendritic cell size decreased, and at the same time, more Mg_2_Ca phases were observed at the grain boundaries, as can be seen in figure 3(a) [43].

Zhang *et al* [23] investigated the Mg–Zn binary alloy system, focusing on the effect of Zn concentration on the microstructure of the as-cast alloy. The content of Zn (in weight) was changed at 1, 2, 3, 4, 5 and 6% into the pure Mg and the microstructure was analysed by x-ray diffraction (XRD). Figure 3(b) shows the microstructure of alloys with different Zn concentrations. When Zn is 1 wt %, the microstructure was purely alpha-Mg. As the Zn content was increased up to more than 4 wt %, the microstructure changed to smaller particle like second phases. With a further increase of Zn, the lamellar eutectics appeared and they were relatively coarse and mostly distributed in grain boundaries as compared to their presence in the inter-dendrite. The same researchers studied the effect of Ca content on the Mg–4.0Zn alloy (i.e. ternary alloy) to see how a third element affects the microstructure. They found that with the increase of Ca more than 0.5% into the Mg–4.0Zn alloy, a second phase of polygonal particles transformed into a eutectic lamellar structure with round particles. The transformation of the ternary alloy was found to be very similar to that of a binary alloy. By adding Zn and Ca, the tensile strength and the elongation were increased from 105 to 225 MPa and 4.2% to 17%, respectively. Furthermore, *in vitro* tests of the alloy showed an elevated corrosion potential with a controlled rate of degradation.

### 3.2. Mechanical properties

**Figure 4.**
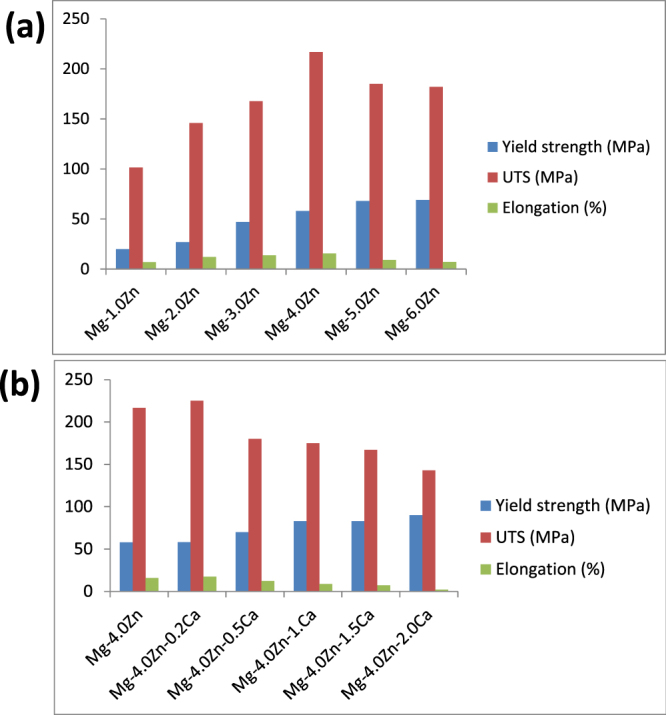
Mechanical properties of (a) Mg–xZn alloys (b) Mg–4.0Zn–xCa alloys (reproduced from [23], copyright 2011 Zhang B P, Wang Y, Geng L. Published in [23] under CC BY 3.0 license. Available from: http://dx.doi.org/10.5772/23929).

Similar to the microstructure, the content of the alloying element consequently affects the mechanical properties of Mg-alloys. Zhang *et al* [23] reported that an increase in Zn content in a pure Mg alloy improved the mechanical properties such as yield strength, ultimate tensile strength (UTS) and elongation (see figure 4(a)). For instance, as the Zn content was increased up to 4.0 wt.%, the mechanical properties were at their highest, exhibiting a yield strength of 58.1 MPa, UTS of 216.85 MPa and elongation of 15.8%. Interestingly, after the peak, the mechanical properties declined. A reduction of the stacking fault energy in the alloy was reported to be responsible for the degradation of the properties. On the other hand, when up to 0.5 wt.% Ca was added into Mg–4.0Zn alloy, the mechanical properties of the ternary alloy increased or remained constant, showing UTS of 215 MPa and elongation of 17.5%, as can be seen in figure 4(b). A further increase of Ca content caused a decline in the mechanical properties. For example, at Ca of 2 wt.% into Mg–4.0Zn, UTS and elongation were found to be 142 MPa and 1.7%, respectively. It was reported that a higher concentration of Ca caused generation of pearl-shaped brittle fractures in the surface, hence degrading strength and elongation. Therefore, it is important that the addition of Ca and Zn into Mg-based alloys must be kept within a certain limit so that the material has a level of ductility exhibiting an increase in flow stress under deformation. Hassel *et al* [14] and Drynda *et al* [44] also studied the effect of Ca content and reported that the tensile strength of the alloy did not improve when the content of Ca was above 0.2 wt%. They further reported that the workability decreased and the force required for extrusion increased with the increase of Ca of about 4 wt.%. Temperature controlled processing was, thus, required for the alloys with higher Ca content. However, hot cracking may result if the deformation temperature during processing matched with the melting temperature of the eutectic phase of the alloys.

### 3.3. Corrosion properties

Mg or its alloy has a unique electrochemical characteristic leading to low corrosion resistance. Studies show that Mg-based implants exhibit improved biocompatibility and corrosion resistance and can accelerate the generation of a hard callous at fractured sites [45]. As compared to traditional biopolymer based implants, they offer higher mechanical strength, as close as the bone’s strength. When Mg-based implants are inserted in physiological environment with a large amount of chlorides, the dissolution process of Mg undergoes the following chemical reaction

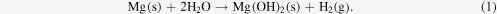



Mg reacts with the water content of human bodily fluid and generates magnesium hydroxide and hydrogen. It is found that magnesium hydroxide acts as a stable protective layer on the implant when the pH value of the bodily fluid is greater than 11.5. However, it accelerates the corrosion of Mg alloys when the pH is lower than 11.5. As pH in bodily fluid is about 7.5 or even lower, the protective magnesium hydroxide layer dissolves and the implant surface is constantly exposed to the chloride-containing fluid. Chloride ions then react with pure Mg or Mg (OH)_2_ to form more soluble MgCl_2_ and facilitate dissolution of Mg(OH)_2_, hence decreasing the protective areas and promoting further dissolution of Mg. The chemical reactions of the underlying accelerated corrosion process are shown below

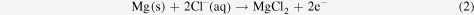


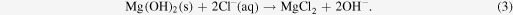



Furthermore, the high content of buffering agents in the bodily fluid is responsible for the accelerated dissolution of Mg. For instance, when Mg is exposed to an aqueous (H_2_O) solution, the following reaction takes place [46]

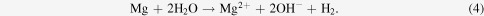



The buffering agents consume the OH^−^ quickly in turn, expediting the conversion from Mg to Mg^2+^. In addition, inorganic substances, proteins and amino acids impact the degradation process. Consequently, Mg-based implants lose the necessary mechanical integrity before the tissue has sufficient time to heal completely. It is reported that hard-tissue repair typically requires implantation of the fixture for at least 12 weeks [9]. As a by-product of Mg dissolution, hydrogen gas is produced and hydrogen evolution equals the magnesium implant corrosion rate. Therefore, the measurement of hydrogen is used as a tool to assess the long-term degradation performance of the Mg-implant. It is to be noted that typically 1 g of magnesium generates 1.081 L of hydrogen gas [14, 47].

Therefore, the control of corrosion rate is important for improving the biomechanical performance of biodegradable Mg/Mg-based implants. Over the years, different approaches were taken to adjust the corrosion rate. They are alloying elements and surface treatments, which include surface coatings and mechanical processing. While the alloying approach shows improvements, several factors which impact overall use and performance of the developed alloy need to be addressed and carefully justified. They are the use of a minimum number of alloying elements, their non-toxicity and biocompatibility. For instance, aluminium and zinc were alloyed with Mg to accelerate the oxidation process while rare earth elements such as cerium (Ce) minimised the oxidation rate of the alloy [47]. Surface treatment approaches have recently been shown to provide excellent improvements in the corrosion performance of Mg-alloyed implants, hence enhancing the bone healing process.

This article will review and highlight the recent developments of surface treatments used to control the corrosion process of Mg-based implants.

## 4. Surface treatments for the control of corrosion rate

### 4.1. Coatings

**Figure 5.**
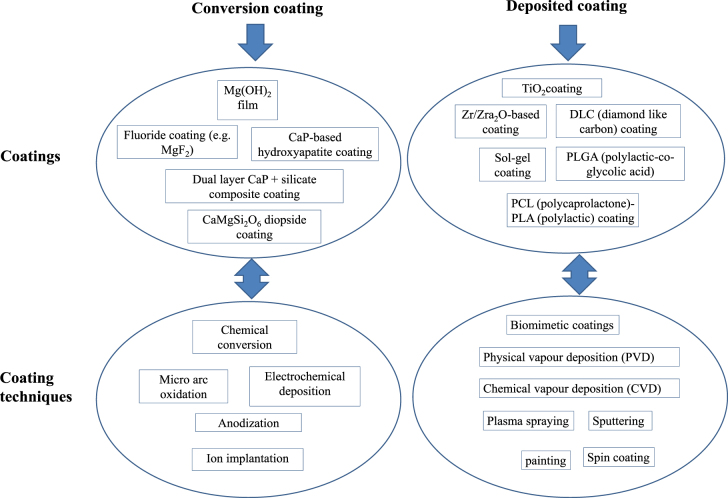
Classification of coatings and their corresponding processing techniques.

A variety of coatings are being investigated to protect Mg/Mg alloy surfaces from severe corrosion. There are two types of coating preparation: conversion and deposition. Conversion coatings are generally developed via chemical or electro-chemical reactions between the substrate and the external coating material or the environment. On the other hand, deposited coatings are mostly metallic based coatings and are widely known in, for example, varnishing in the automotive industry. In some cases, a conversion layer is developed on the substrate as a pre-treatment before applying the final coating to increase the adhesion strength. While the conversion process is used in many applications such as automotive industries, the deposition process is a widely used and accepted process for biomedical coatings, particularly, in orthopaedic implants, as outlined in [48]. Figure 5 illustrates the different types of coatings in terms of conversion and deposition, showing the key example coatings and their corresponding processing techniques used for controlling the corrosion performance. It must be noted that the prime goal of the coatings on Mg-based alloy implants is to improve the corrosion resistance, in addition to possessing other important properties such as biocompatibility, bioactivity, and antibiotic ability.

In the following sections, we will highlight important innovative coatings and their production techniques recently studied by various researchers in adjusting the corrosion rate or the degradation rate of Mg-based alloys. The main focus is to review the efficacy of various coatings applied onto the Mg/Mg-based alloy surface in improving its corrosion resistance via *in vitro* as well as laboratory based studies.

#### 4.1.1. Fluoride coatings

**Figure 6.**
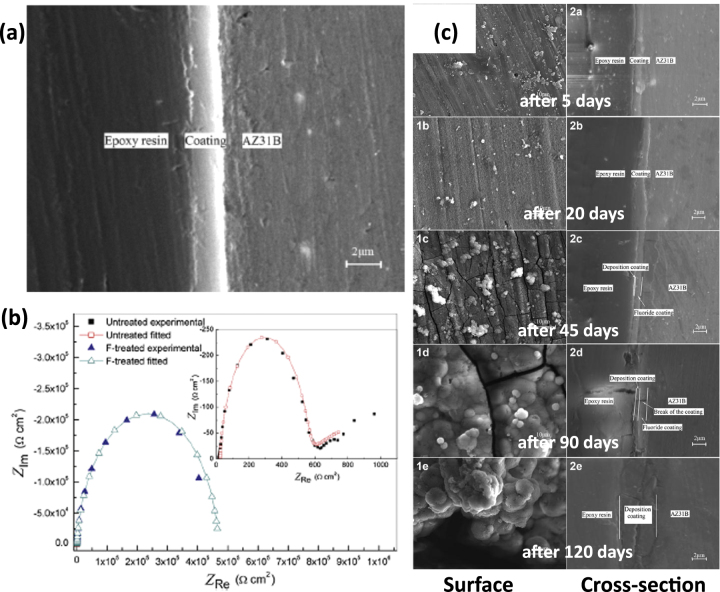
(a) Scanning electron microscopy (SEM) image of fluoride coated AZ31 sample (b) electrochemical impedance spectroscopy (EIS) spectra of bare and fluoride coated sample in simulated blood plasma (c) surface and cross-sectional morphologies of fluoride coated sample in simulated blood plasma at different time intervals (reprinted from [51], copyright 2014, with permission from Elsevier).

Drynda *et al* [44] developed a magnesium fluoride (MgF_2_) coated Mg-Ca alloy, in which MgF_2_ was deposited onto Mg–Ca alloy, and tested the corrosion performance in a saline (e.g. NaCl) solution. The degradation rate of the alloy was evaluated by measuring hydrogen generation. It was reported that the MgF_2_ coated alloy degraded slowly as compared to the uncoated alloy (Mg–Ca alloy) and no hydrogen gas was detected from 8 to 40 h. Thomann *et al* [49] applied the same coating on a Mg–Ca0.8 alloy and found that the fluoride coating on the Mg–Ca implants slowed down the corrosion rate and retained higher mechanical properties for a long period of time (even after 6 months) than its uncoated counterpart. Similar effects of fluoride coatings on Mg-alloys in terms of improving corrosion and mechanical integrity performance were examined and reported by other researchers [50]. Figure 6 shows surface morphologies and electrochemical impedance spectroscopy (EIS) spectra results for uncoated and fluoride-coated AZ31 samples in a simulated blood plasma [51]. It is clear that magnesium fluoride coatings have been successful in modifying magnesium-based implants by both *in vitro* and *in vivo* assessments, and become a potential candidate for biomedical applications. However, it must be noted that caution must be taken as hydrofluoric acid typically used for MgF_2_ coating generation may be harmful to operators as well as ensuing environments. Fluoride salt can be a potential alternative in this case, which requires further comprehensive investigations to confirm its use and benefits.

#### 4.1.2. Mg(OH)_2_ films by alkaline treatments

**Figure 7.**
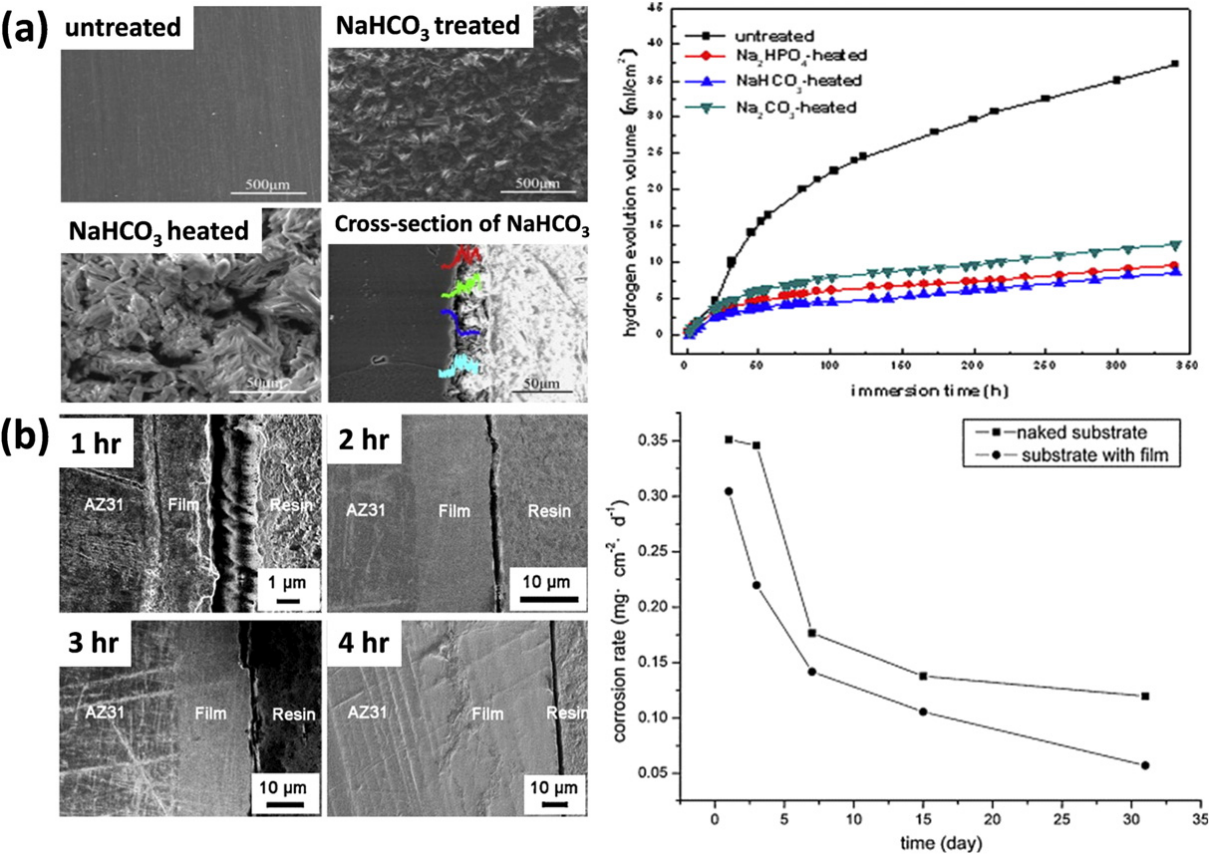
(a) SEM images of untreated and NaHCO_3_ treatments of Mg–Ca alloy (left) and H_2_ evolution of different alkaline treatments of Mg-Ca alloy (right) (reprinted from [52], copyright 2009, with permission from Elsevier) (b) SEM images of cross-section of alkaline (NaOH) treated Mg–Ca1.4 alloys for different time intervals (left) and corrosion rate of bare and treated sample (right) (reprinted from [54], copyright 2011, with permission from Elsevier).

In an aqueous environment like human bodily fluid, the Mg alloy forms Mg(OH)_2_ which acts mainly as a protective layer and prevents corrosion. Alkaline treatments of Mg-alloys are thus used to create more of this layer on the implant surface. Gu *et al* [52], Li *et al* [53], and Zhu *et al* [54] studied the effect of alkaline heat treatments on Mg–Ca alloys. For instance, Gu *et al* [52] reported that after soaking the Mg–Ca alloy in three alkaline solutions (Na_2_HPO_4_, Na_2_CO_3_, and NaHCO_3_) for 24 h and heat treating at 500 °C for 12 h, the alloy underwent a slower degradation rate (figure 7(a)). This is due to the thicker magnesium oxide films generated on the surface by alkaline treatments, in which the thicknesses of oxide layers on the Mg–Ca alloy substrate formed by Na_2_HPO_4_, Na_2_CO_3_, and NaHCO_3_ treatments, are 13 *μ*m, 9 *μ*m and 26 *μ*m, respectively. Furthermore, cytotoxicity tests revealed that none of the alkaline heat-treated Mg–Ca alloys induced toxicity in cells. In another study by Zhu *et al* [54], the AZ31 alloy was treated with 5.66 wt.% NaOH solution at 160 °C to generate a protective Mg(OH)_2_ film. Tests performed in Hank’s solution for up to 4 h showed that the corrosion rate of the treated alloy was inhibited effectively (figure 7(b)). They also found no effect on the cell growth or changes in cell morphology due to the additional oxide films.

#### 4.1.3. Calcium phosphate (CaP) coating

**Figure 8.**
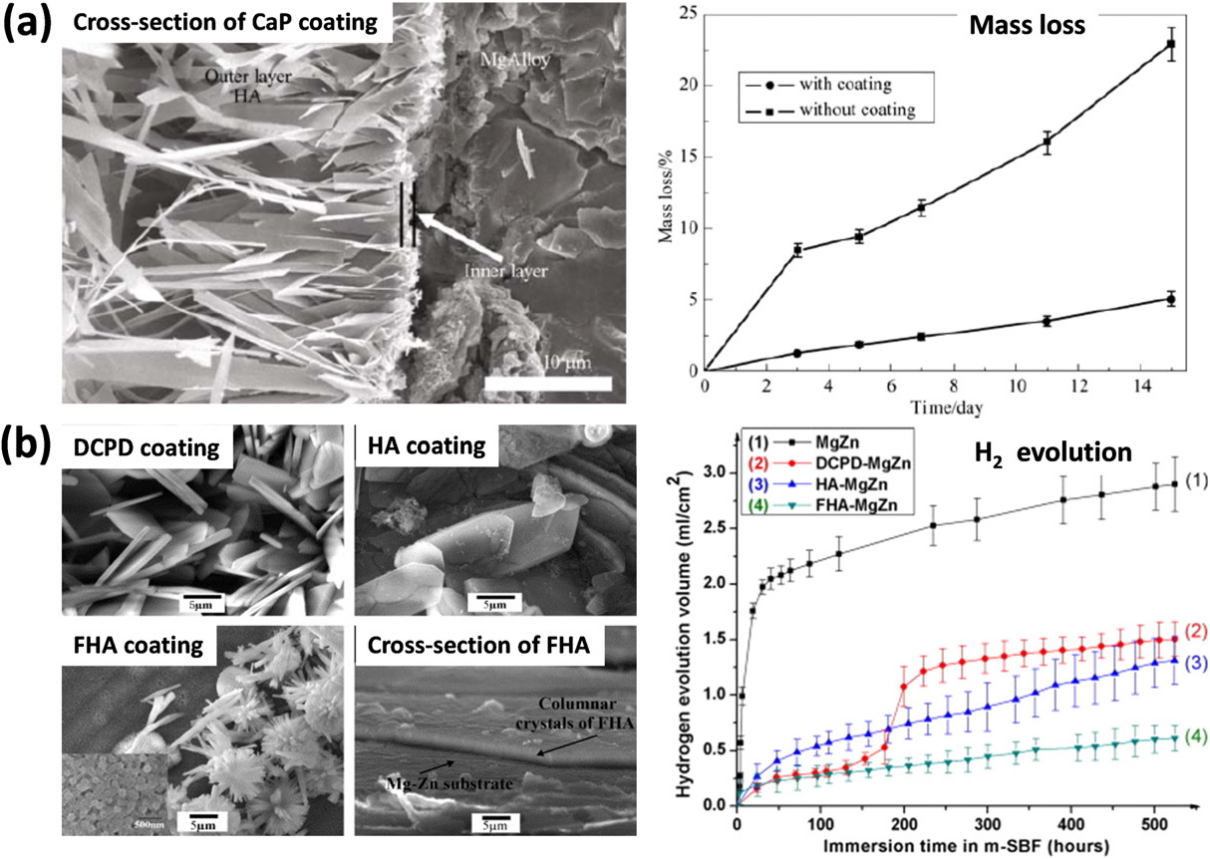
(a) SEM image of cross-section of CaP coating on AZ31 alloy and its mass loss, i.e. corrosion rate in NaCl solution (reproduced from [13], copyright 2008, with kind permission from Springer Science and Business Media). (b) SEM images of dicalcium phosphate dihydrate (DCPD), hydroxyapatite (HA) and fluoridated hydroxyapatite (FHA) coatings on Mg–Zn alloy and their corrosion performances (i.e. H_2_ evolutions) (reprinted from [55], copyright 2010, with permission from Elsevier).

As an organic coating, CaP-based coating is of the highest interest and investigated to enhance the corrosion resistance of magnesium and magnesium alloys. There are many methods used to produce CaP coatings on the surface of a Mg-alloy. For instance, Cui *et al* [13] applied CaP coatings on a Mg–Ca1.0 alloy using electrochemical deposition. After performing electrochemical tests in Hank’s solutions, CaP coated Mg–Ca alloy exhibited an increase in corrosion resistance as can be seen in (figure 8(a)). Further, the coated sample revealed higher hydrogen evolution rate than the uncoated sample. However, the corrosion rate increased as the pits were generated in the coatings. CaP coating was also shown to be beneficial for biocompatibility. However, the CaP layer formed on Mg–Ca alloy surface was an amorphous mixed (Mg, Ca)-phosphate which can be carbonated and hydrated [45].

#### 4.1.4. CaP-based hydroxyapatite coating

CaP-based hydroxyapatite coatings are widely used in biomedical implants due to their chemical and structural similarities with bone materials. For instance, Song *et al* [55] developed hydroxyapatite (HA, Ca_10_(PO_4_)_6_(OH)_2_) and fluoridated hydroxyapatite (FHA, Ca_5_(PO_4_)_3_(OH)_1−*x*_F*_x_*) coatings on a Mg–Zn alloy and assessed their corrosion performance in simulated body fluid (SBF). HA and FHA coatings were found to delay the corrosion rate and promote the nucleation of osteo-conductive minerals for one month (figure 8(b)). Notably, as can be seen in figure 8(b), compared to HA coating, FHA showed more stable and improved corrosion resistance. Similar improvements in terms of cell growth and osteointegration were reported in a previous study by Li *et al* [56]. Furthermore, Kannan and Orr [57] studied the effect of potentiostatically alkaline treated hydroxyapatite coating on a AZ91 alloy and the results suggested that the corrosion resistance and biomechanical integrity can be improved by 20% using low cathodic voltage hydroxyapatite coatings, as compared to the uncoated samples.

#### 4.1.5. TiO_2_ coating

**Figure 9.**
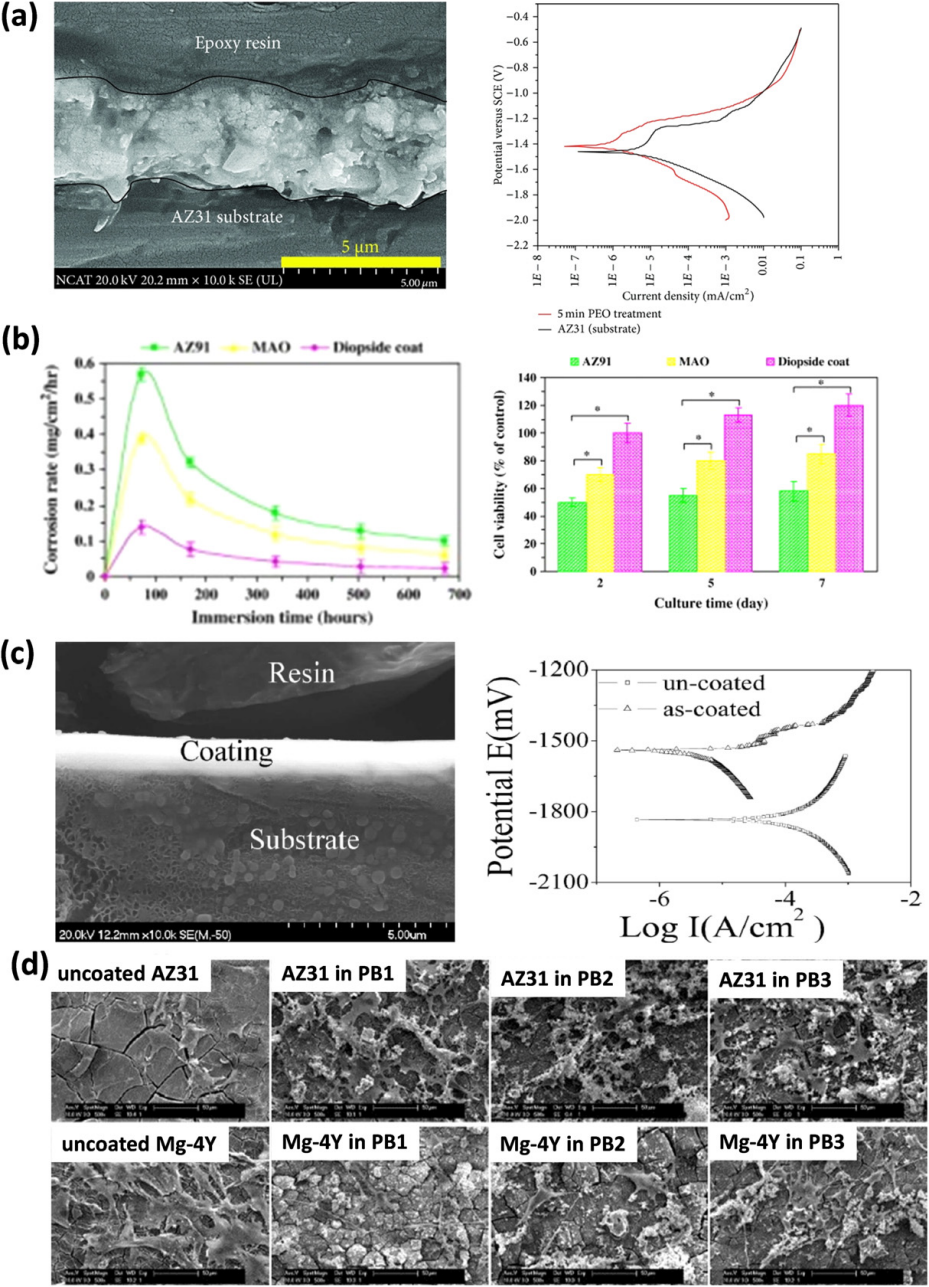
(a) Cross-section morphology of TiO_2_ coated AZ31 substrate and its potentio-dynamic polarization curve with respect to the uncoated substrate (reproduced from [59] under a Creative Commons Attribution License), (b) corrosion rate and cell viability of CaMgSi_2_O_6_ diopside coating along with uncoated and micro-arc oxidation (MAO) coated AZ91 substrate (reprinted from [60], copyright 2014, with permission from Elsevier), (c) cross-sectional morphology and potentio-dynamic polarization curves of Zr/ZrO_2_ coated AZ91 substrate (reproduced from [61], copyright 2011 Cambridge University Press), (d) scanning electron microscopy (SEM) images of morphologies of CaP + silicate coated AZ31 and Mg-4Y alloys immersed in polymyxin B1 (PB1), polymyxin B2 (PB2), and polymyxin B3 (PB3) after 3 days of culture (reprinted from [62], copyright 2011, with permission from Elsevier).

A coating made of nanoparticles of TiO_2_ was applied to a Mg–Ca alloy to increase the corrosion resistance. Li *et al* [58] and White *et al* [59] studied the effect of TiO_2_ coating on the corrosion performance. For example, Li *et al* [58] reported that a TiO_2_ coated Mg–Ca alloy showed delayed corrosion as compared to the uncoated alloy surface. Further, the coated alloy exhibited three times lower corrosion current density than the uncoated counterpart. Similarly, White *et al* [59] prepared and applied a TiO_2_ thin layer on AZ31 Mg alloy and tested the sample in NaCl solutions. XRD and SEM evaluation of test samples indicated that the oxide films of MgO and Mg_2_TiO_4_ generated due to TiO_2_ coating were uniform, porous and compact, which significantly improved the corrosion resistance as compared to the fresh AZ31 sample (figure 9(a)). However, the application of TiO_2_ onto a Mg-alloy needs to be further investigated to increase the catalytic activity and to ensure the coated alloy is biocompatible and environmentally safe for its dedicated use in biomedical implants.

#### 4.1.6. CaMgSi_2_O_6_ diopside coating

Razavi *et al* [60] prepared and applied a CaMgSi_2_O_6_ coating on the AZ91 Mg alloy. In this study, using micro-arc oxidation (MAO) they first created a rough and porous structure on the Mg substrate, which provided satisfactory sites for depositing nanostructural CaMgSi_2_O_6_ diopside powders and increasing interfacial bonding strength. In the next step, they deposited CaMgSi_2_O_6_ diopside powders using an electrophoretic deposition (EPD). Electrochemical evaluation in SBF environment showed that the diopside-coated magnesium alloy improved the corrosion resistance and *in vitro* bioactivity (figure 9(b)). For instance, after immersion in SBF for 72 h, the corrosion rate of pure AZ91 and diopside-coated samples was 0.57 and 0.14 mg cm^−2^ h^−1^, respectively (see figure 9(b)). In particular, the initial degradation rate for AZ91 in first 72 h was 0.08 mg cm^−2^ h^−1^, which was pretty much similar to the one investigated by Song *et al* [18]; furthermore, this indicated that the results of immersion tests were consistent with those of electrochemical tests. Razavi *et al* [63] also studied akermanite (Ca_2_MgSi_2_O_7_) coating on Mg alloys and found a similar improvement by the coating in terms of reducing the ecorrosion rate and preserving biocompatibility.

#### 4.1.7. ZrO_2_ coating

As a bio-inert ceramic material, zirconia-based thin layers are widely applied on the surface of implants. It facilitates fixing the implant with the surrounding tissues without producing any chemical or biological bonds. It is reported that a bone-like apatite is produced on the surface of zirconium layer, which is responsible for the improved interfacial strength between the bone and the implant. Previously many researchers studied the effect of Zr and ZrO_2_ based coating on Mg alloy to adjust the corrosion rate [18, 61, 64–66]. For instance, using cathodic arc deposition process, Xin *et al* [61] developed a 1.5 *μ*m thick Zr–ZrO_2_ dual layer on the AZ91 Mg alloy and tested its mechanical and corrosion behaviour in SBF. Results showed that the Vickers hardness of the coated sample was increased by about 88% as compared to the uncoated alloy substrate. Immersion and electrochemical tests (for up to 18 h) show that the corrosion resistance of the coated sample increased significantly (figure 9(c)) while electrolyte penetration was found to damage the protective coating layer after long exposure in SBF. Song *et al* [18] applied a compact and uniform Ni–P–ZrO_2_ composite coating on AZ91D magnesium alloys, in which at first a Ni–P layer was deposited on the Mg alloy substrate and then another layer made of ZrO_2_ nanoparticles was made on the Ni–P layer. Electrochemical evaluation indicated that the Ni–P–ZrO_2_ coating exhibited better corrosion resistance than that of Ni–P coating due to additional ZrO2 together with Ni–P. In addition, the Ni–P–ZrO_2_ coating enhanced hardness and wear resistance as compared to the uncoated sample. In fact, the initial Ni–P layer provided solution and precipitation strength while ZrO_2_ facilitated dispersion hardening into Ni–P, making the overall composite coating harder than the Ni–P layer alone.

#### 4.1.8. Dual layer CaP + silicate composite coating

Dual layer inorganic composite coatings were studied recently by researchers to adjust the corrosion rate of Mg-based alloys [62, 67]. Using an aqueous phosphating bath method, Singh *et al* [62] deposited homogeneous films of CaP and silicate on two magnesium alloy systems, AZ31 and Mg-4Y. *In vitro* and bioactivity tests, in which the samples were immersed in polymyxin B1 (PB1), polymyxin B2 (PB2), and polymyxin B3 (PB3) (polymyxin—a mixture of several closely related polypeptides) for up to 3 days of cell culture, showed that the silicate-based CaP coatings decreased the degradation of AZ31 while they increased the degradation of Mg-4Y (figure 9(d)). In addition, silicate–CaP coatings were observed to increase cell attachment on the AZ31 alloy. Further studies reported that silicon-substituted CaP had the ability to retain osteoclasts over a short period of time and increased the strength of the bond between the bone and implant as compared to pure CaP coated samples [68].

### 4.2. Coating techniques

Depending on the coating materials and types, different coating formation techniques are employed on Mg alloys to improve their corrosion resistance and biocompatibility. In particular, coating techniques are selected based on whether it is substrate-involving coatings, non-substrate-involving coatings and composite coatings [69].

#### 4.2.1. Chemical conversion treatment

The chemical conversion approach is generally applied to form substrate-involving coatings. In this case, the thin layers are developed *in situ* via the chemical reaction between the Mg or Mg alloy substrate and the treatment agent. A detailed review of chemical conversion treatments is presented by Chen *et al* [70], and it is suggested that proper pre-treatment to functionalise the surface is crucial to determine the overall coating performance. Mg(OH)_2_ and fluoride based coatings are two of the most widely used coatings developed by chemical conversion treatments, and are shown to increase the corrosion resistance along with no signs of induced toxicity of surrounding cells.

For instance, Gu *et al* [52] studied alkaline-based coatings, in which the magnesium substrate was soaked in three alkaline solutions (NaHCO_3,_ Na_2_HPO_4_ and Na_2_CO_3,_) and heat treated at a certain temperature. Electrochemical and cytotoxicity tests showed that all the coated samples decreased the corrosion rate and revealed no signs of toxicity. Ultrapure water, hydrofluoric acid, phytic acid and phosphor were also employed as the conversion treatment agents to form coatings which eventually reduced the corrosion rate and facilitated the cell growth surrounding the implant [49, 69, 71]. Further, rare earth elements such as Ce and Nd were used to form the conversion layers on Mg-based alloys. It was reported that a small amount of rare earth material was not detrimental to human health. Cui *et al* [72] and Levy and Aghion [73] developed Ce and Nd-based diffusion coatings on magnesium alloy substrate (e.g. AZ31) and found that both coatings enhanced the corrosion resistance significantly. However, caution must be taken about whether these rare earth materials may pose any toxicity when employed over a longer period of time. Chemical conversion coating is still considered a cost-effective technique, and therefore it is important to make use of the method to wider applications within the biomedical field.

#### 4.2.2. Micro-arc oxidation (MAO)

MAO, often also called plasma electrolytic oxidation (PEO), is another widely used technique to form porous and robust thin coatings with high adhesion strength onto magnesium and its alloys. The typical chromate coating is no longer available due to a ban imposed by EU regulatory authorities. MAO has been regarded as the most commercially viable route for developing corrosion protective layers on Mg/Mg-based alloys. MAO consists of an electrolytic bath, a working electrode consisting of an electrically connected Mg alloy and a stainless steel counter-electrode. The quality of the produced layer heavily relies on the bath, alloy properties, and processing parameters, e.g. reaction time, applied voltage [48]. CaP and ceramic based coating such as silicate and ZrO_2_ are typically developed by using MAO. It is found that MAO fabricated coating works as an intermediate layer for depositing nano-sized HA coating because it can produce pinning force when HA is deposited in the pores. Per Gao *et al* [74], the MAO coating was fabricated under a pulse voltage mode, and the cell potential was increased gradually up to 165–175 V and then the working electrode was oxidised for 30 min. The electrolyte was prepared by using a range of solution mixtures. For instance, Gu *et al* [75] prepared the electrolyte from the solution of 10 g L^−1^ sodium silicate with 3.5 g L^−1^ sodium. In another study by Shi *et al* [76], MAO was performed in 500 mL aqueous solution containing 50 g L^−1^ NaOH, 40 g L^−1^ Na_2_SiO_3_, 20 g L^−1^ Na_2_B_4_O_7_ and 40 g L^−1^ Na_3_C_6_H_5_O_7_ at a constant potential of 90 V for about 140 min. However, the disadvantages with MAO process are that surface pores and cracks formed during MAO accelerate the corrosion rate in a chloride-rich physiological environment. Further, the main phase of coating is MgO which is often not suitable for effective cell growth.

#### 4.2.3. Electrochemical deposition (ED)

ED, a non-substrate-involving coating technique, is one of the most recognised and widely used coating formation methods for biodegradable magnesium and its alloys. Though the process is mainly concerned with the deposition of inorganic phases, the coating produced can be achieved via both purely deposition and, to some extent, the conversion at the interface. It has the capability of forming uniform coatings, controllability of maintaining the required thickness and chemical composition of the coating, and most importantly, low deposition temperature [45]. Cathodic ED, a traditional process, is commonly used to deposit CaP based on coatings. However, HA coatings made by cathodic ED are sometimes fragile and less stable, hence degrading its long term corrosion resistance. Therefore, a modified pulse electrodeposition was recently employed to fabricate CaP coatings. Using this technique, Wang *et al* [77] developed Ca-deficient HA (Ca-def HA) coating on a Mg–Zn–Ca alloy and found that both corrosion resistance and mechanical properties such as ultimate tensile strength (and time to fracture) were improved by the Ca-def HA coatings. It was also shown that, compared to traditional cathodic electrodeposition, the pulse electrodeposition process assisted more effectively in precipitating magnesium, calcium and oxide ions, which created a strong, uniform and compact protective layer, thus preventing the magnesium alloy substrate from further corrosion. Further, Wen *et al* [78] and Kannan and Orr [57] studied multi-step ED processes to produce HA-based coatings on AZ31 Mg alloys, in which alkaline treatments using NaOH solution produce a fine microstructure. Electrodeposition is then used to modify or transform the microstructure from platelets to nanowiskers or rod-shaped particles. *In vitro* evaluation indicates that the coating produced by the modified ED process enhances mechanical strength significantly.

#### 4.2.4. Anodisation

Anodisation is another coating technique which uses the concept of classical electrochemical conversion, in which a film having a thickness of ranging from 5–200 *μ*m is developed on the substrate material. During anodisation, the metal (of coating material) as anode is transformed into oxide film with controlled corrosion protective, decorative, and functional properties. As reported by many researchers, the anodising performance of Mg and its alloys generally depends on a range of key parameters of the process. They are: electrolytic composition (e.g. aqueous or non-aqueous solutions), applied constant voltage or current, substrate types, quality of Mg and alloying element concentrations [45]. In order to further improve the anodisation performance, self-ordering electrochemical processes have been studied. The benefit of the process is that it can produce nano-structured oxide films from aqueous and non-aqueous solutions, and even under certain optimized conditions, a nano-tubular porous structure is created on the substrate material. However, it was reported that Mg alloys facilitated in forming nano-structured oxide films in non-aqueous solutions rather than aqueous solutions [79]. The degree of quality of the corrosion resistant oxide layers depends on the anodisation parameters. For example, appropriate adjustment of the process parameters such as electrolytic concentration, current density, and anodisation time, affects the quality of coating structure, including the density of porosity and crystallinity [48]. While anodisation has a significant potential in creating corrosion resistive oxide layers, the process may need to be further investigated to explore and determine the optimized parameters which will able to develop a well-defined and structured coating, thus enhancing corrosion resistance, cell growth (i.e. interaction between cells and films) and biocompatibility.

#### 4.2.5. Ion implantation

**Figure 10.**
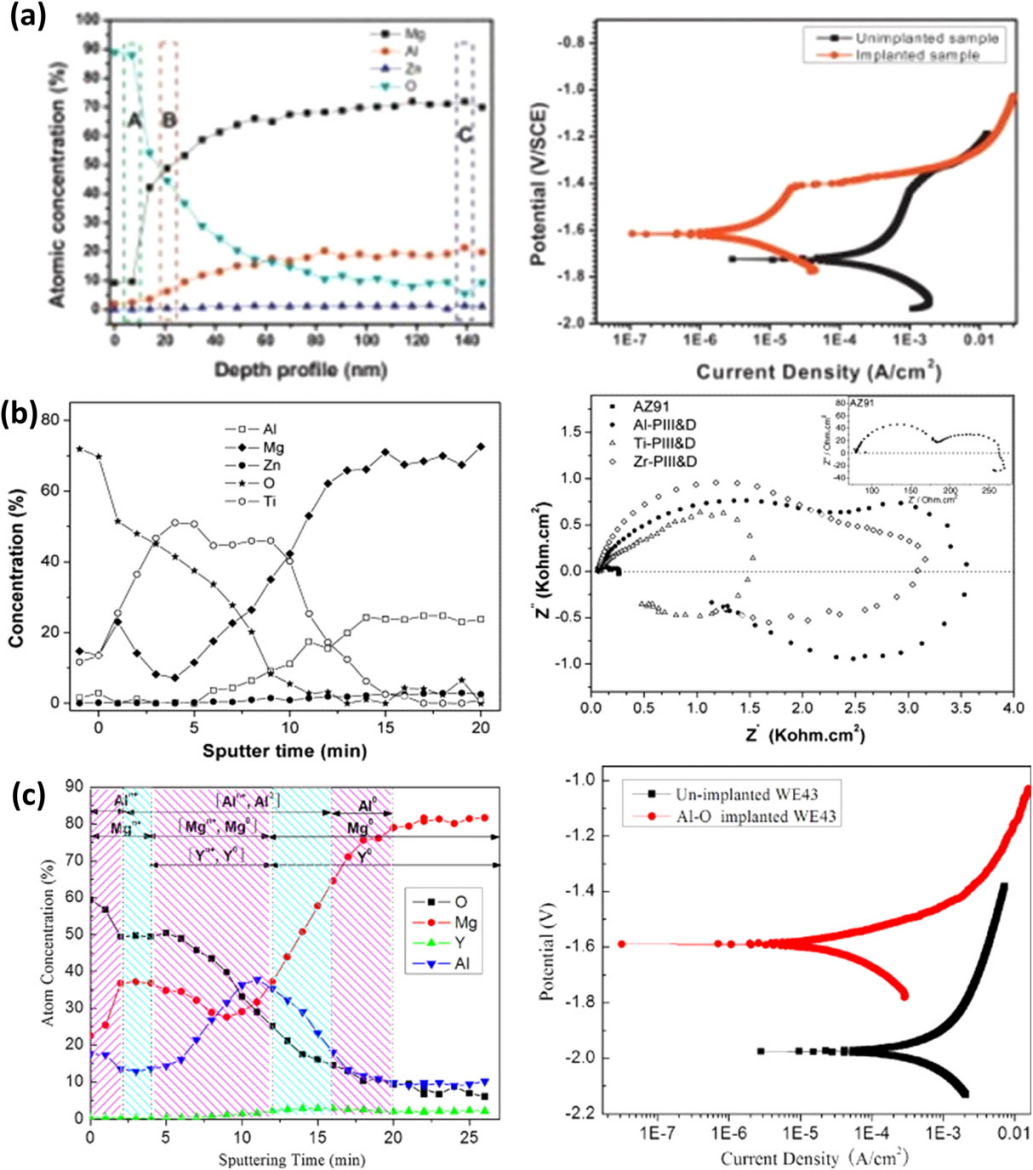
(a) X-ray photoelectron spectroscopy (XPS) depth profiles of oxygen-implanted AZ31 alloy, and potentiodynamic polarization curves of unimplanted and ion-implanted alloy in SBF (reprinted from [84], copyright 2013, with permission from Elsevier), (b) XPS depth profiles of Ti-PIII&D implanted AZ91 alloy and Nyquist plots of as-received and PIII&D samples in SBF (reprinted from [85], copyright 2007, with permission from Elsevier), (c) XPS depth profiles of WE43 alloy after Al and O plasma ion implantation, and polarization curves of unimplanted and ion-implanted alloy in SBF (reprinted from [86], copyright 2012, with permission from Elsevier).

To further improve the corrosion resistance, Mg and its alloys have been ion implanted as reported by previous research [48]. Ion implantation involves a process in which ions are accelerated and impinged into the modified surface. As opposed to the surface coatings techniques described above, as an advanced technique, the ion implantation process introduces ions into the substrate without requiring any electrolytic solutions. Further, an ion-implanted layer does not have an abrupt interface and hence potential layer fracture or delamination is not likely to occur [69]. As the process only can produce a very thin layer of the modified surface, generally the corrosion resistance is not adequate in the long term [16, 80, 81]. In another study by Wu *et al* [82], ion implantation was used a pre-treatment process to improve the adhesion strength between a Ti coating and the AZ31 substrate. Depending on the implanted elements, ion implantation for Mg modification is categorised into three types—gas ion implantation, metal ion implantation, and dual ion implantation. Organic elements such as oxygen, nitrogen, and carbon dioxide gases are implanted into Mg and its alloy substrates and the surface modification is found to improve the corrosion resistance of the alloy (see figure 10(a)) [83, 84]. While gas ion implantation creates an oxygen or nitrogen-rich layer on the substrate, metal ion implantation introduces metal elements to form surface alloying into magnesium and its alloy substrate. Al, Zn, Zr, Ti and Ta are few metallic elements which are often ion implanted into magnesium alloys (e.g. AZ31, AZ91) [16, 85]. The modified layer with metal ion implantation enhances the corrosion resistance (see figure10(b)). The mechanism for improved corrosion resistance is ascribed to the formation of intermixed layer consisting of an outer oxide layer (of MgO or Mg(OH)_2_), an intermediate metallic layer and a bottom layer rich in metallic elements [85]. While these elements are found to be individually biologically friendly to the human body, further investigations are needed to ensure their biocompatibility when the composite layer consisting of multiple metals are implanted. In order to develop an oxygen rich metal oxide layer, metal and oxygen dual ion implantation has been employed to achieve better corrosion resistance than either gas ion implantation or metal ion implantation used individually. For instance, Zhao *et al* [86] used Al and O ion implantation to create an Al_2_O_3_ protective layer and found that the modified layer delayed the degradation of the substrate (figure 10(c)). Similar improvements were observed for Ti–O, Zr–O, and Cr–O dual ion implanted surfaces studied by other researchers [87–89]. Furthermore, the detailed description of different types of ion implantation can be found in a comprehensive review performed by Tian and Liu [69]. Despite its unique benefits as outlined above, the ion implantation technique is generally costly and often may not be suitable for intricate geometries of components such as implants and porous scaffolds.

#### 4.2.6. Biomimetic coatings

**Figure 11.**
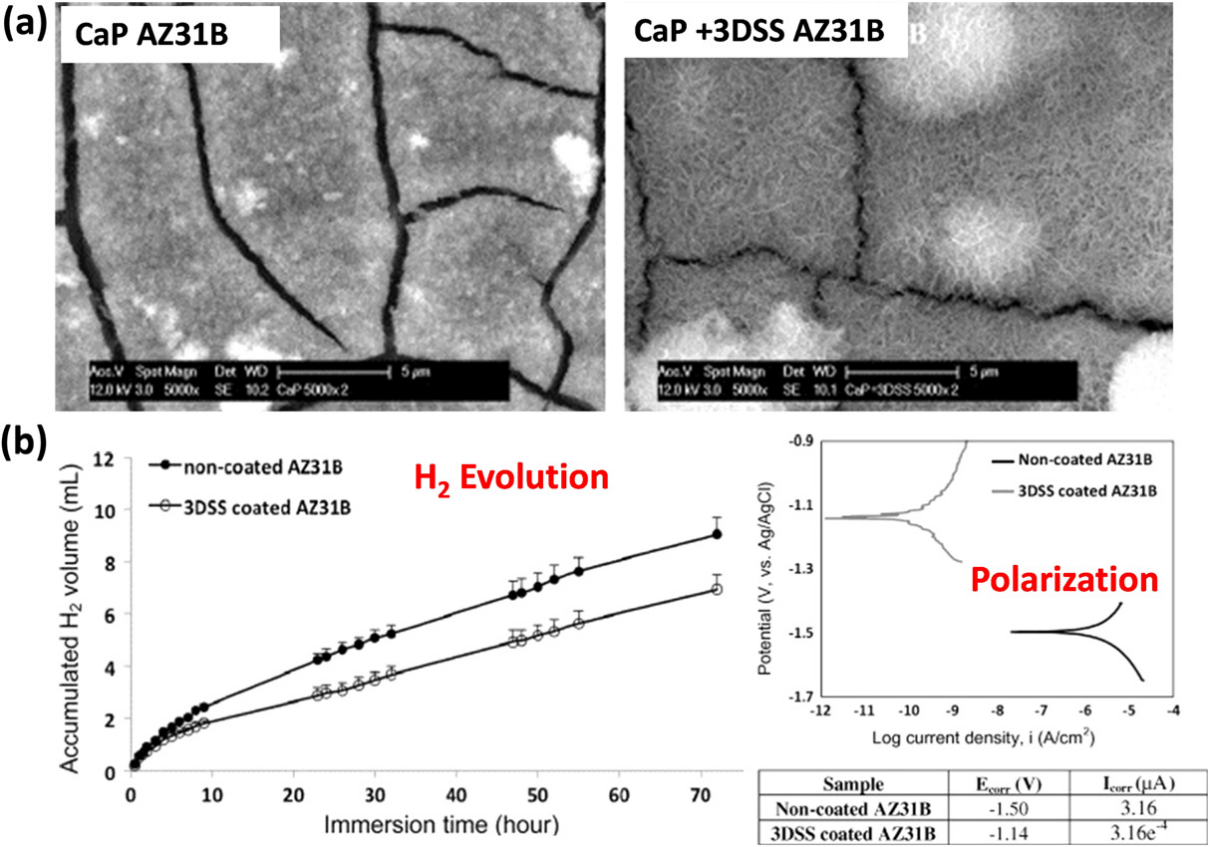
(a) surface morphologies of CaP coating on AZ31B alloys with and without the co-presence of dentin sialophosphorprotein (3DSS) peptide (b) H_2_ generation and polarization profiles of non-coated and 3DSS coated AZ31B alloy (reprinted from [90], copyright 2013, with permission from Elsevier).

While traditional coating techniques, including chemical conversions, electrochemical treatment and ion implantation as described above, are widely studied, the results in improving the corrosion resistance are variable and often lack the bioactive properties necessary for controlling cellular behaviour [90]. Biomimetic coating is a novel and promising way to significantly enhance corrosion resistance and biocompatibility. It is a biologically derived coating which provides cell signalling capabilities for better tissue generation. For instance, Cui *et al* [90] comprehensively developed biomimetic coatings based on highly acidic bone protein, known as dentin sialophosphorprotein (DSPP), on a Mg alloy and investigated their corrosion and biological performance. DSPP is a non-collagenous extracellular matrix (ECM) protein found in bone and teeth while phosphophoryn (PP) is a cleavage product of DSPP which provides dentine mineralization and cell signalling in bone and dentine. As technical isolation of PP protein is quite difficult because of rapid degradation, the same authors [13] followed the principles of bio-mimicry and developed a biomimetic coating based on a peptide motif using the amino acid sequence of PP. The peptide is composed of three repeats of DSS (Asp–Ser-rich) amino acids. The study is focused on testing the feasibility of coating biomimetic peptides onto a Mg alloy (AZ31B), examining the reduction in corrosion rate and assessing whether the peptide induces CaP precipitation onto the alloy substrate. SEM analysis showed that 3DSS peptide coating on AZ31B was smooth and compact while polishing marks were evident on non-3DSS coated surfaces (see figure 11(a)). Immersion tests in a SBF solution showed that hydrogen volume generation from the 3DSS-coated AZ31B alloy was found to be consistently lower than that from the non-coated alloy. The corrosion potential for the 3DSS-coated sample was 400 mV higher than the non-coated one, while the corrosion current density (of 3.16 × 10^−4^ *μ*A) was significantly lower than the uncoated sample (3.161 *μ*A) (figure 11(b)), all of which indicated that 3DSS biomimetic peptide coatings were stable and corrosion resistant. Further, it was reported that the 3DSS peptide-induced CaP coating on AZ31B alloys showed a larger and more organised crystal-like structure as compared to that without the facilitation of 3DSS biomimetic peptide.

#### 4.2.7. Physical vapour deposition (PVD) coating

VD is one of the well-known and commercially employed coating techniques, in which, a protective layer is deposited on the substrate. Due to its capability in producing highly-dense nano-structured coatings, the technique is widely used to deposit a hard coating layer on the substrate material to improve wear and corrosion resistance. As compared to electro-chemical coating processes such as MAO, the PVD technique can produce thick and strongly adhering layers on the substrate. Studies have shown that the corrosion can be affected by the coating material selection and process parameters [91]. Magnetron sputtering also enables the formation of metal alloys that are not accessible via conventional processing techniques [92]. This so called vapour quenching process has the potential to generate a corrosion resistive coating on Mg alloys for which the process has not been previously studied. In the past, using the magnetron sputtering method, many studies explored the application of hard coatings on Mg/Mg-based alloys to improve corrosion resistance as well as wear resistance, in which the typical multi-layer coatings used are TiN, CrN, TiAlN, TiO_2_, Al_2_O_3_ and DLC [93–95]. For instance, Altun and Sen [96] developed multi-layer AlN and AlN+TiN on a AZ91 magnesium alloy using PVD DC magnetron sputtering and reported that the multilayer coating increased the corrosion resistance significantly. Researchers studied the growth and characteristics of a dense nano-structure biocompatible CaP-based HA coating deposited using a PVD technique on orthopaedic implant surfaces [97, 98]. Most of the recent studies focused on depositing the sputtered HA coating on the surface of metallic implants including titanium/titanium alloys, NiTi, stainless steel, and silicon plates [98–100]. It was reported that the HA films deposited on such metallic surfaces using RF magnetron sputtering exhibit a highly dense and nano-composite structure, providing good biocompatibility and mechanical integrity [101]. Recently, Surmeneva *et al* [102] employed the RF magnetron PVD technique to deposit CaP based HA coating on bioabsorbable AZ31 magnesium alloys and reported an increase in the wear resistance and hardness of the alloy. They found that by applying a negative bias (−50 V) on the substrate during deposition process reduces the grain and crystallites size, hence further increasing the hardness of the coating.

In order to reduce the acceleration of galvanic corrosion between the substrate and protective coating produced by PVD, Hoche *et al* [103] studied DC magnetron sputtering (DCMS) and high power impulse magnetron sputtering (HiPIMS) to produce a coating of TiMgN with rare earth elements such as Y and Gd on a AZ31hp Mg alloy. It was shown that compared to the DCMS and TiMgYN coating, the TiMgGdN coating with HiPIMS technique exhibited excellent corrosion resistance. As compared to DCMS, HiPIMS was found to increase the grain refinement, hence reducing corrosion initiation sites such as pinholes, clusters, and droplets. On the other hand, the addition of Gd into TiMgN causes the formation of dense and stable oxide films, which prevent galvanic corrosion at the interface between the coating layer and the substrate. In a similar study, Xie *et al* [104] investigated the gradient duplex coating combining nitrogen ion implantation and AlN–MoS_2_–phenolic resin produced by the RF magnetron sputtering method and reported that the AlN interlayer, together with ion implantation, significantly improves the tribo-corrosion resistance of the Mg alloy. While ion implantation creates more dense oxide films, AlN–MoS_2_ retards the galvanic corrosion between the coating and the substrate. While grain refinement is shown to improve the corrosion resistance, other factors such as surface free energy and residual stress within the protective oxide films may impact the corrosion performance [105], which is still considered to be a topic of interest and needs to be further investigated. Plasma anodisation together with RF magnetron sputtering was applied to produce hard coatings such as CrN and Al_2_O_3_ [106], and the corrosion behaviour was investigated at macroscopic and microscopic levels. In this case, at first a 0.05 *μ*m thick plasma anodised layer is deposited on the substrate, and the process is similar to the anodisation in an aqueous electrolyte by replacing the electrolyte with oxygen plasma. The final step is to apply a relatively thick (4–5 *μ*m) layer of hard coating by conventional magnetron sputtering.

It is therefore clear that the performance of the coating developed by PVD (e.g. magnetron sputtering) depends not only on the choice and integration of appropriate coating materials but also on the coating process conditions including power pulse and bias. As a potential technique, PVD can thus be employed to produce a stable, strong coating which can delay the degradation of orthopaedic implants made of Mg alloys. A hybrid coating process combining RF/DC magnetron sputtering together with additional ion implantation or plasma anodisation would potentially further increase the corrosion resistance of the alloys. Along with proper choice of coating materials, the process parameters including applied pulse power and bias negative voltage, are critical factors impacting the coating performance. Further research should focus on how PVD can robustly be applied to produce CaP HA and biomimetic coatings, which are considered potentially new and accepted coating materials for the control of corrosion process in Mg alloy-based implants.

### 4.3. Mechanical processing

As described above, while the application of coatings on Mg and its alloys has shown potential improvements in increasing corrosion resistance, they still suffer from biocompatibility problems due to the addition of different new coating elements and the corresponding processing requirements. Further, the coating process is often time consuming and quite expensive. As an alternative to the coating approach, mechanical surfacing treatment of the near surface or bulk material is considered a promising and potentially cost effective and efficient technique to enhance the bio-corrosion performance. As the process does not deal with any additional new materials except for the original base substrate material (i.e. Mg alloys), there is no issue with biocompatibility. A unique advantage of the mechanical treatment over the coating process is that it improves the biomechanical integrity of the material in terms of increased ultimate strength and fatigue resistance. The following sections will highlight the recent developmentsion various mechanical treatment approaches employed in controlling biocorrosion and mechanical performances of Mg and its alloys.

#### 4.3.1. Shot peening

**Figure 12.**
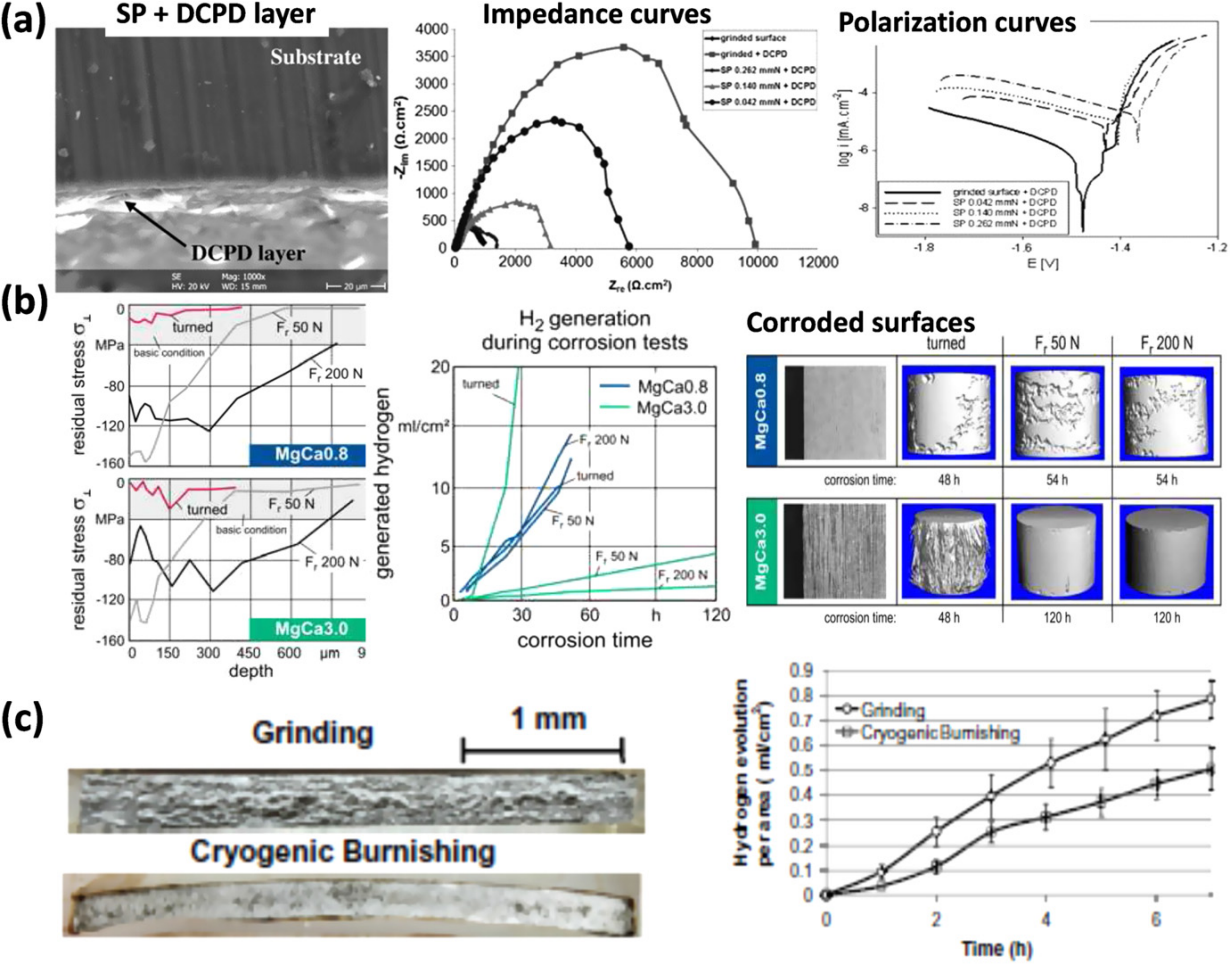
Surface morphologies of short peened and dicalcium phosphate dihydrate (DCPD) coated AZ31 alloy and the corresponding impedance curves with various surface treatments (reprinted from [113], copyright 2014, with permission from Elsevier), (b) residual stress on Mg–0.8Ca and Mg–3.0Ca alloys gendered by turning followed by deep rolling, their H_2_ generation rate and the corresponding mass loss at different time intervals (reproduced from [115], copyright 2011 Leibniz Universität Hannover, IFW. Published in [115] under CC BY-NC-SA 3.0 license. Available from: http://dx.doi.org/10.5772/22793), (c) cross sectional surface morphology of cryogenic + severe plasticity burnished AZ31 alloy after immersion for 200 h and its corresponding corrosion resistance (i.e. H_2_ generation) (reprinted from [116], copyright 2011, with permission from Elsevier).

As a simple and inexpensive method, shot peening (SP) is widely used to improve the fatigue and the corrosion performance of structural metallic alloys [107–109]. SP is a cold surface work hardening process which introduces near surface compressive residual stress into the material to prevent fatigue crack initiation and growth. With an appropriate choice of SP process parameters, fatigue performance can be improved significantly. For instance, Liu *et al* [110], Wagner *et al* [111], and Fouad *et al* [112] have applied SP on Mg-based alloys, e.g. Mg–10Gd–3Y, AZ31 and AZ80, and ZK60 alloy, and reported that the surface modifications with SP generated effective compressive stresses which shifted the fatigue crack nucleation site from the surface to subsurface regions, thus improving the overall fatigue strength of the material. Recently, Mhaede *et al* [113] applied SP with ceramic shots (Z850, Ø850 *μ*m) on the AZ31 magnesium alloy followed by the electro-deposition of a calcium phosphate coating, e.g. dicalcium phosphate dihydrate (DCPD). Microhardness and surface roughness were measured for mechanical assessment while electrochemical tests were performed to evaluate the corrosion performance. Results showed that the hardness of the SP sample increased from 55 up to 101 HV0.025 at a surface depth of 0.20 mm. DCPD coating on the SP surface showed higher surface roughness as compared to SP-only surface. It was reported that the DCPD coating formed on the SP sample was relatively dense and adherent to the surface, which potentially improved the mechanical strength of the coating (see figure 12(a)) and high surface roughness implied a positive result in terms of osseointegration. Polarization resistance of the SP substrate decreased 22–71 times as compared to the fresh substrate (i.e. only ground surface), meaning that SP caused an increased surface energy and induced more residual stresses, thus making the surface more active. However, polarization resistance of the SP + DCPD coating was higher than those without DCPD coating (see figure 12(a)). Furthermore, the SP + DCPD coated sample exhibited a higher corrosion resistance as compared to the ground + DCPD coated sample. This clearly indicates the impact of SP in combination with the coating in controlling the corrosion resistance of Mg-based alloys.

#### 4.3.2. Machining

Tool-based material removal, i.e. machining, is another mechanical surfacing process to improve corrosion resistance by generating a high finish on machined Mg-based implants. Von der Höh *et al* [114] employed three different surface treatments on Mg–0.8Ca alloy implants and investigated their effect on the *in vivo* degradation behaviour of the alloy for a 3–6 month period. The implant surfaces were mechanically processed with turning, cylinder threading, and sand blasting, in which medial roughness was 3.65 *μ*m for the turned sample and 32.7 *μ*m for the sand-blasted one. Different cell growth or bone formation around the implant and hydrogen gas generation were considered as tools to evaluate the degradation process. It was shown that sand-blasted samples degraded the fastest as compared to turned and threaded ones. On the other hand, the turned surface exhibited the lowest structural loss, showing hardly any hydrogen gas formation. In addition, after 3 months the turned implants showed the best bone–implant integration as compared to sand-blasted and thread surfaces. The highest amount of fibrotic tissue along with hyaline cartilaginous was found to grow around the turned sample, as compared to threaded cylinder and sand-blasted implants. It is quite apparent that a smooth surface with fine finishing (i.e. lower surface finish) by the turning process is favourable for achieving osteointegration between the bone and the Mg-based alloy implants.

Using the turning process, Denkena and Lucas [117] attempted to control the degradation rate of a Mg–3.0Ca alloy via modifying the surface integrity including surface roughness and residual stress. They studied the effect of different process parameters on the generation of compressive residual stress into the sub-surface, which were likely to improve the corrosion resistance. It was reported that higher cutting forces due to low cutting speed induced higher and deeper compressive stress and hence decreased the corrosion rate, while a fine surface finish at the same condition was observed to have a negligible impact. Later on, Denkena *et al* [115] investigated the application of both turning and milling processes along with deep rolling to adjust the *in vitro* corrosion performance of Mg–0.8Ca and Mg–3.0Ca alloys. Turning followed by deep rolling at a higher force (of 200 N) can induce a significant amount of compressive stress at higher surface depth of 800 *μ*m (figure 12(b)). Milling with a relatively larger cutting edge radius of insert is able to introduce larger compressive stress at higher surface depth as compared to the turning process. While the surface and subsurface properties (i.e. residual compressive stress) from mechanical processing are approximately similar for both Mg–3.0Ca and Mg–0.8Ca alloys, the corrosion behaviour of two differs significantly (figure 12(b)). As can be seen from figure 12(b), turned and deep-rolled Mg–0.8Ca generated hydrogen at the same rate, while for the Mg-3.0Ca alloy, the deep -rolled sample showed a significant reduction of hydrogen generation, thus delaying degradation of the alloy. This further indicates that deep rolling after the turning process reduces the corrosion rate for the Mg–3.0Ca alloy. This can be attributed to higher and deeper compressive stress into the subsurface, which closes the superficial micro-pores and stops the formation and propagation of cracks (see figure 12(b)).

#### 4.3.3. Low plasticity burnishing or deep rolling

Low plasticity burnishing, analogous to deep rolling, is a new mechanical treatment process used to improve the corrosion and the fatigue resistance of metallic alloys. It produces cold work hardening and introduces minimal plastic deformation generating compressive residual stress into the subsurface. Low cold working offers both thermal and mechanical stability of the beneficial compression, and the process has recently been used in aerospace and biomedical industries [109, 118, 119]. Similar to low plasticity burnishing, as already indicated, Denkena and Lucas [117] employed deep rolling to develop adaptable degradation profiles for the purpose of different medical applications of Mg–Ca alloys. Both processes can introduce higher and deeper compressive stress as high level of cold working associated with the process often degrades the mechanical stability of compressive layer generated. Interestingly low plasticity burnishing typically requires less cold working and the technique with appropriate processing parameters would, therefore, be a promising way to improve the biomechanical stability. Compared to shot peening, the benefit of using low plasticity burnishing or deep rolling is that the processed surface does not become contaminated with any external material and hence third body wear is unlikely to occur [120]. Salahshoor and Guo [15, 17] studied the process mechanics of ball burnishing and low plasticity burnishing and investigated the effect of different process parameters on improving the corrosion resistance and the mechanical surface integrity of Mg–Ca alloys.

Very recently, unlike low plasticity burnishing, severe plasticity burnishing integrated with cryogenic cooling has been employed to enhance the corrosion resistance of Mg–Al–Zn alloys [116] and compared the performance with traditional grinding process. Cryogenic burnishing produced a fine-grained microstructure at a relatively larger depth, inducing severe compressive stress as well as generating a fine surface finish (figure 12(c)). Hydrogen evolution tests in NaCl solutions suggested that cryogenic burnishing reduced the corrosion rate by almost 50% as compared to the ground-only sample (see figure 12(c)). The above results clearly imply that that burnishing or deep rolling as a simple, cost-effective mechanical process would be able to offer a unique opportunity to remarkably enhance the functional performance of metallic materials, such as the corrosion resistance of Mg-based alloys, by tailoring their microstructures and introducing residual stress into the subsurface.

Overall, it seems that burnishing or deep rolling would be an efficient way to mitigate corrosion-induced failure of the implants without changing either the material or design of the implants. While shot peening may negatively influence the corrosion resistance, burnishing could be potentially employed to control both bio-corrosion and mechanical integrity, and hence further research works are needed to achieve the maximum benefits. It has been reported that it is possible to generate the depth of compression into the surface, which may exceed the maximum corrosion pit depth in implants, and thus save the implants from cracking or pitting-induced failures.

## 5. Future challenges and directions

The key to developing biodegradable Mg alloy orthopaedic implants is in how to adjust their corrosion rate and biomechanical integrity in a highly chloride-rich human physiological environment. The main aim is that implants would remain in the human body and maintain sufficient mechanical integrity over a certain period of time, and then be absorbed by human cells when the bone tissues heal completely. In addition to the alloying technique, surface modifications, including coatings and mechanical treatments, were widely studied and investigated over the decades to develop Mg alloy-based implants with controlled degradation and improved biomechanical strength required for the bone to recover sufficiently. Coatings with new and innovative materials reduce the corrosion rate and offer osseo-integration between the bone and the implant; however, they may not be the complete solutions and often develop the biocompatibility problems when used as implants. Mechanical processing seems a promising technique without introducing further toxicity problems, while exhibiting an enhanced corrosion resistance and mechanical strength including fatigue and ultimate strength. Both approaches have advantages and disadvantages and pose challenges that need to be addressed to completely realize the benefits of biodegradable Mg-based alloys in orthopaedic applications.

### 5.1. Coatings

The current literature reveals that a wide range of coating materials and coating formation techniques are employed to enhance the corrosion resistance of Mg-based alloys. Among all coatings, calcium phosphate and organic based coatings are of the greatest interest to many researchers. However, there are a number of factors which impact the performance of the underlying coatings. Surface chemistry, which influences the stability of phases and adhesion strength in coatings, is an important parameter. In the case of chemical conversion coatings, the coating pre-treatment seems to be more crucial than the choice of coating formation process to be applied because it helps functionalise the substrate and thus facilitates the coating process to develop a stronger and more stable coating, particularly in aqueous solutions. Due to the wide variation of the techniques (e.g. immersion tests, polarization studies) and the associated parameters (composition and concentration of electrolytes) used for corrosion evaluation, it is often difficult to compare and assess the performance of the coatings. For instance, the electrochemical test or potentio-dynamic polarization is typically conducted in SBF or NaCl solution, in which the corrosion current density is measured as an indicator of the corrosion rate. It is reported that for a corrosion test in a given electrolytic solution such as SBF, the reduction in corrosion rate is varied [48] and this indicates that the corrosion rate is controllable.

Most of the *in vivo* or *in vitro* studies in the literature investigated the corrosion behaviour of the coated samples over a period of maximum of a few days or weeks. The results from the studies may not be used to determine the long-term corrosion performance of the same sample. Therefore, advanced and stable coatings have to be developed by considering their thickness, adhesion and morphology, and *in vitro* corrosion tests need to be conducted over a long period of time to understand the long-term efficacy of the coatings. As the coatings will disappear with time, the corrosion process will accelerate, which will reduce the mechanical integrity of the implant before the complete healing of the bone. Therefore, in addition to the coating material itself, the selection of Mg alloy substrate, along with the phase, microstructure and geometry of the sample material, is crucial to understanding and determining the true corrosion performance of the alloy implants. In addition, most of the past work deals with the assessment of corrosion performance for a shorter period of time (e.g. few days or months), which is not sufficient to determine the life-time performance of the alloy implant. Therefore, it is imperative that a wider range of experimental data consisting of *in vitro* and *in vivo* evaluation of longer-term corrosion performance is undertaken to deliver a greater understanding about the underlying degradation behaviour of the coated Mg alloy surfaces.

The uniformity of the corrosion process is another important aspect, which implies a well-defined degradation rate and dissolution behaviour of the coated samples. It is reported that coated samples often possess defects such as pores and cracks at which corrosion first starts to initiate. This leads to a non-uniform corrosion rate in nature and further accelerates fracture and flaking off of the coatings as the dissolution propagates further.

In spite of its high adhesion strength and enhanced corrosion resistance in a shorter period of time, PEO coating does not exhibit satisfactory corrosion resistance over a longer period of time due to its porous surface structure including potential cracks. Therefore, it is important to minimise the porosity of the coating by adjusting the coating process parameters or by performing pre-treatments before the application of the coating which is often done for organic-based coatings. This will increase the adhesion and bonding strength of the coating required to control the corrosion rate as well. In addition, the PEO coating suffers from a lack of surface biocompatibility for potential cell proliferation [69]. Hence further research needs to be devoted towards improving biocompatibility. Magnetron sputtering PVD would be a potential method to produce a dense, deep, and stable corrosion resistance coating. In such a case, in addition to its capability of enhancing corrosion resistance, surface modification by ion implantation is considered a promising way to improve biocompatibility despite the fact that the modified layer is very thin.

The surface topography of the coating is another important aspect of the coated samples that may influence the corrosion rate. While many previous studies focused on the observation of the coated surface, no correlation between surface roughness, morphology and corrosion behaviour was described. Furthermore, in order to achieve an optimised biological performance, the design and fabrication of the coatings should be tailored for its specific applications such as stents, orthopaedic implants. For instance, a coating with a porous surface is often not suitable for stents. Composite coatings with the choice of appropriate biocompatible materials and thickness of layers would be a potential approach to further enhance the corrosion resistance. The coatings can be prepared in a way so that different layers would provide different functionalities in an orderly manner, so that the required corrosion resistance, mechanical integrity and biocompatibility are maintained throughout the service life of the coatings (i.e. during effective dissolution process in human body). However, the bonding strength between the layers, and between the layer and the substrate, should be sufficient so that it does not negatively affect the corrosion rate. New and advanced plausible coating techniques, along with new biomaterials, thus need to be researched and developed. For instance, a biomimetic coating with a composite structure having intermediate layers designed each for specific functionality such as corrosion resistance, biocompatibility, and cell growth, would be a potential candidate in this regard.

### 5.2. Mechanical treatments

Alternative to the coatings, mechanical treatments such as shot peening, machining and burnishing or deep rolling, are a simple, cost-effective and non-toxic tools which can enhance the corrosion resistance. The combined effect of cold work hardening and the near-surface compressive residual stress prevent the nucleation and the propagation of cracks, hence improving the fatigue strength and the corrosion resistance. Compared to shot peening, burnishing (or deep rolling) with low plastic deformation can introduce higher and deeper compressive stress into the subsurface, thus increasing the delay of degradation of Mg alloys. On the other hand, while severe plastic deformation during cold working often damages the protective compression layer, cryogenic cooling with liquid nitrogen improves the protective layer. In order to further improve the corrosion resistance, various process parameters such as speed, feed rate, rolling force, roller diameter and lubricant need to be optimised. Novel techniques such as hybrid treatment by combining the coating and mechanical processing would be a promising solution to adjust the corrosion and mechanical bio-integrity required for implants in a physiological environment. In addition to coatings, deep compressive layers within subsurface, which will enhance corrosion resistance and mechanical integrity, can be further augmented by new and improved mechanical treatments such as synergetic machining-burnishing together with cryogenic cooling. Optimization of the process parameters is another important factor which needs to be taken into consideration to attain the best possible performance of mechanical treatment. Overall, more extensive research efforts have to be conducted to discover and yield the expected outcomes of these potential techniques.

## 6. Concluding remarks

This current literature review demonstrates that Mg and Mg-based alloys are an emerging and promising candidate for the development of biodegradable, biocompatible orthopaedic implants. However, high degradation and the loss of biomechanical integrity due to fast corrosion rate often limit the application of these alloys. Over the years, researchers have studied and employed a range of techniques to mitigate the problem. It has been revealed from the analysis of the literature that the microstructure, mechanical properties and corrosion resistance can be improved by chemical alloying processes. However, the alloying often poses biocompatibility problems due to the potential toxicity of new additional alloying elements, and is found to be experimentally tedious and expensive. Besides alloying, surface treatment including coating and mechanical processing, is shown to be a promising means to tackle high degradation kinetics of magnesium based alloys in a chloride-rich human physiological environment. A wide range of coatings, including organic or inorganic, is developed and studied, which improve the corrosion resistance. The appropriate coating material, coating formation technique, process parameters and corrosion testing method need to be decided to obtain an accurate and useful improvement in terms of reduced degradation kinetics of coated Mg-based alloys. A calcium phosphate-based coating is commonly used and studied; however, it still lacks sufficient stability in the longer term due to potential crack formations. A biomimetic coating with amino peptic motif and biofunctional organic molecules as outer layers is another new means to control corrosion rate as well as to facilitate cell behaviour to achieve specific bio-functions. However, more research effort is required to assess the efficacy of the approach, including its potential future adoption in orthopaedic implants. The *in vitro* and *in vivo* investigations presented in this review show that surface modification by mechanical treatments such as burnishing and deep rolling, can not only increase the corrosion resistance but also improve the mechanical properties, such as the fatigue life, of the implant. Mechanical treatments so far are found to be a more emerging area for the development of Mg alloy-based implants in future as it is cheap, simple and does not introduce any toxicity problems. It must be noted that while there have been tremendous number research works performed over the decades to evaluate the use of Mg-based alloys as orthopaedic and cardiovascular implants, there is still a strong need to continue investing research effort to fully and successfully realise Mg-based alloys as orthopaedic materials in biomedical device industries. This should include the design and development of a new paradigm of coatings, mechanical processing techniques, and complete characterisation of critical factors including corrosion rate, surface chemistry, near surface residual stress, and coating topography. A hybrid technique combining mechanical processing and coating, along with drug eluting polymeric layers [121], would be a promising means to control the corrosion rate, hence maintaining the desired mechanical integrity of the implants as they heal and remodel adequately. All of these are expected to potentially widen the applications of Mg-based alloys in revolutionising the successful development of biomedical devices such as bone fixations and stents.
